# Global trends in COVID-19 Alzheimer's related research: a bibliometric analysis

**DOI:** 10.3389/fneur.2023.1193768

**Published:** 2023-06-05

**Authors:** Chenjun Cao, Sixin Li, Gaoya Zhou, Caijuan Xu, Xi Chen, Huiwen Qiu, Xinyu Li, Ying Liu, Hui Cao, Changlong Bi

**Affiliations:** ^1^Department of Psychiatry, School of Clinical Medicine, Hunan University of Chinese Medicine, Changsha, Hunan, China; ^2^Department of Psychiatry, Hunan Brain Hospital (The Second People's Hospital of Hunan Province), Changsha, Hunan, China; ^3^Department of Neurology, School of Clinical Medicine, Hunan University of Chinese Medicine, Changsha, Hunan, China; ^4^Department of Neurology, Hunan Brain Hospital (The Second People's Hospital of Hunan Province), Changsha, Hunan, China; ^5^Department of Neurosurgery, Xiangya Hospital, Central South University, Changsha, Hunan, China; ^6^National Clinical Research Center for Geriatric Disorders, Xiangya Hospital, Central South University, Changsha, Hunan, China

**Keywords:** COVID-19, Alzheimer's, bibliometric analysis, CiteSpace, VOSviewer

## Abstract

**Background:**

The COVID-19 pandemic has significantly impacted public health, putting people with Alzheimer's disease at significant risk. This study used bibliometric analysis method to conduct in-depth research on the relationship between COVID-19 and Alzheimer's disease, as well as to predict its development trends.

**Methods:**

The Web of Science Core Collection was searched for relevant literature on Alzheimer's and Coronavirus-19 during 2019–2023. We used a search query string in our advanced search. Using Microsoft Excel 2021 and VOSviewer software, a statistical analysis of primary high-yield authors, research institutions, countries, and journals was performed. Knowledge networks, collaboration maps, hotspots, and regional trends were analyzed using VOSviewer and CiteSpace.

**Results:**

During 2020–2023, 866 academic studies were published in international journals. United States, Italy, and the United Kingdom rank top three in the survey; in terms of productivity, the top three schools were Harvard Medical School, the University of Padua, and the University of Oxford; Bonanni, Laura, from Gabriele d'Annunzio University (Italy), Tedeschi, Gioacchino from the University of Campania Luigi Vanvitelli (Italy), Vanacore, Nicola from Natl Ctr Dis Prevent and Health Promot (Italy), Reddy, P. Hemachandra from Texas Tech University (USA), and El Haj, Mohamad from University of Nantes (France) were the authors who published the most articles; The Journal of Alzheimer's Disease is the journals with the most published articles; “COVID-19,” “Alzheimer's disease,” “neurodegenerative diseases,” “cognitive impairment,” “neuroinflammation,” “quality of life,” and “neurological complications” have been the focus of attention in the last 3 years.

**Conclusion:**

The disease caused by the COVID-19 virus infection related to Alzheimer's disease has attracted significant attention worldwide. The major hot topics in 2020 were: “Alzheimer' disease,” COVID-19,” risk factors,” care,” and “Parkinson's disease.” During the 2 years 2021 and 2022, researchers were also interested in “neurodegenerative diseases,” “cognitive impairment,” and “quality of life,” which require further investigation.

## Introduction

In late 2019, China detected its first pneumonia of unknown cause (1). A new coronavirus has been isolated in China, the severe acute respiratory syndrome coronavirus 2019 (2, 3). Coronavirus Disease 2019 (COVID-19) was then named by the World Health Organization (4). There were some clinical symptoms of COVID-19, such as disturbances of taste and smell (5). Patients with COVID-19 have reported neurological problems and possible neurological invasion (6–8). Infection with COVID-19 can affect Alzheimer's disease, and long-term neurologic conditions such as Alzheimer's disease can develop (9, 10). However, even if a nasal swab tests positive for COVID-19, the patient's cerebrospinal fluid may be devoid of viral particles. The presence or absence of COVID-19 in CSF (cerebrospinal fluid) may depend on the severity of systemic disease and the degree of neurotropic tropism of the virus (11).

COVID-19 infiltrated Alzheimer's dementia research (12). People with Alzheimer's disease (AD) were at higher risk of developing severe acute respiratory syndrome coronavirus 2 (SARS-CoV-2) and its associated morbidity and mortality (13, 14). Older people with Alzheimer's disease and coronavirus-19 virus infection can present with mild, unusual diarrhea or lethargy (15). AD patients' family caregivers were sometimes called “invisible second patients (16).” COVID-19 isolation rapidly increased behavioral and psychological symptoms in ~60% of AD patients, and two-thirds of caregivers experienced stress-related symptoms (17). Boutoleau-Bretonnière et al. reported that confinement exacerbated neuropsychiatric symptoms in patients with cognitively impaired AD but did not induce such symptoms in more cognitively intact patients (18). Lara et al. reported that during the 5 weeks of lockdown, neuropsychiatric symptoms of AD patients worsened, with agitation, apathy, and abnormal motor activity being the most affected symptoms (19). In Italy, caregivers reported significant increases in anxiety (9.18%), depression (6.26%), irritability (2.28%), and distress (9.80%) (20). A wealth of research on Alzheimer's disease and SARS-CoV-2 has been published in the past 3 years. However, studies have yet to comprehensively analyze the impact of AD on SARS-CoV-2 research and propose potential future research directions in this field.

Bibliometrics research was becoming more and more extensive. Bibliometric research can calculate the productivity of institutions, countries, and authors and explore the frequency of keywords that are hot/cutting-edge in a particular field (21, 22). Using the method of bibliographical economics, we can summarize the current situation and development trend of a particular subject or a specific unique disease to put forward the direction and ideas for future research work (23). CiteSpace and VOSviewer are currently the most popular data analysis and visualization research methods (24, 25). Therefore, this article intends to use the method of bibliometric analysis to sort out the relevant research on COVID-19 AD to clarify its knowledge structure and critical issues. In addition, this paper also puts forward several suggestions for future research work.

## Methods and materials

### Search strategy

On February 7, 2023, search query strings TS = (“Alzheimer's^*^” OR “Senile Dementia^*^” OR “AD”) and TS = (“Coronavirus disease 2019” OR “COVID-19” OR “SARS-CoV-2”) against WoSCC were used to identify COVID-19-related publications in Alzheimer's disease research. The document had only articles and reviews and the language was English. This database was collected and organized by Chenjun Cao and Sixin Li, respectively. Disagreements are discussed with the other two people (Ying Liu and Xinyu Li) to unify their opinions.

### Data extraction and analytical methods

We extract these bibliometric parameters [title, keywords, author, institution, country or region, journal, publication year, total number of citations (TC), number of citations per publication (CPP), and cited references] and export them to Microsoft Excel 2021 (Redmond, Washington, USA) and VOSviewer (version 1.6.18, Leiden University) to identify the most influential contributors (lead authors, institutions, and countries). VOSviewer and CiteSpace were two bibliometric software programs. On this basis, VOSviewer can build the author of the article and the subject map of the paper based on the cited information in the article, and it can also build a keyword map based on the coexistence information. This project also provides an observer that enables exhaustive analysis of file metrics graphs. VOSviewer can be displayed in many ways to emphasize various aspects. It has zoom, scroll, and search capabilities for detailed map inspection (25). CiteSpace is software based on Java. The system integrated cluster analysis, social network analysis, and other technologies and was a visual analysis software for scientific and technological document data with substantial application value. Innovation was mainly reflected in the deep mining of co-citation information in scientific and technological papers, the analysis of the knowledge structure of relevant knowledge fields, the analysis of the development trend and correlation of scientific and technological literature, and the analysis of the intermediary role between critical nodes in scientific and technological literature. The model can explore a time-varying career planning process from the research field to the knowledge base; it will present this information as a colored map (26). They use literature, keywords, authors, cooperative institutions, and countries as the primary research objects, using mathematical statistics and other research methods for quantitative analysis (27). VOSviewer and CiteSpace (version 6.1.R6) present the collaborative map and intensity of collaborations between authors, institutions, and countries to demonstrate their impact on Alzheimer's disease SARS-CoV-2 research.

Additionally, keyword and reference clustering were used to capture knowledge in the domain. The project will also display possible research frontiers using the keyword co-occurrence of VOSviewer and CiteSpace. A relationship diagram between VOSViewer and CiteSpace shows that the size of the nodes represents the number of publications, while the lines indicate the links between them. A larger node represents more published articles, and a wider line represents a closer connection between two nodes.

## Results

### General data

**Figure 1:**
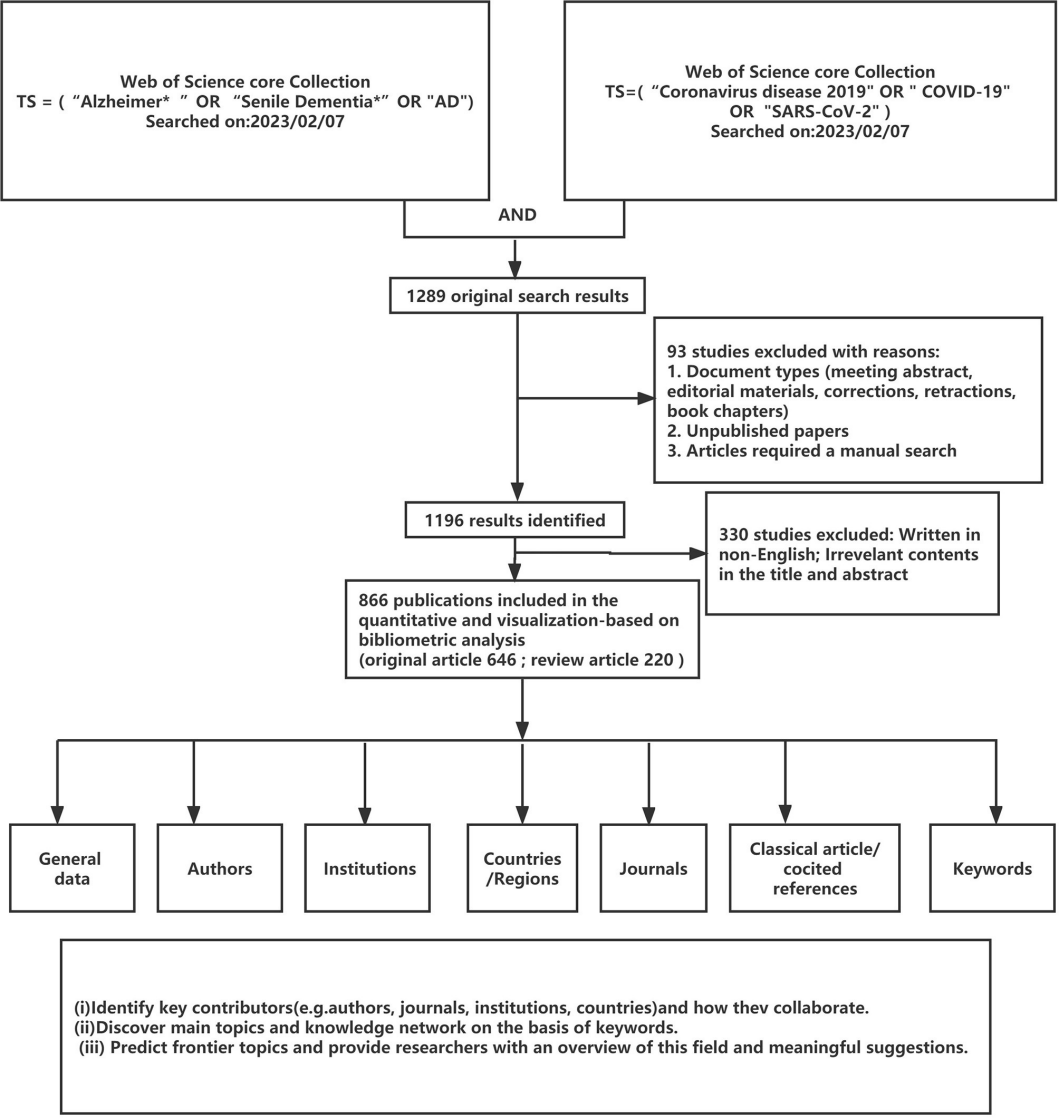
Flow chart.

**Figure 2:**
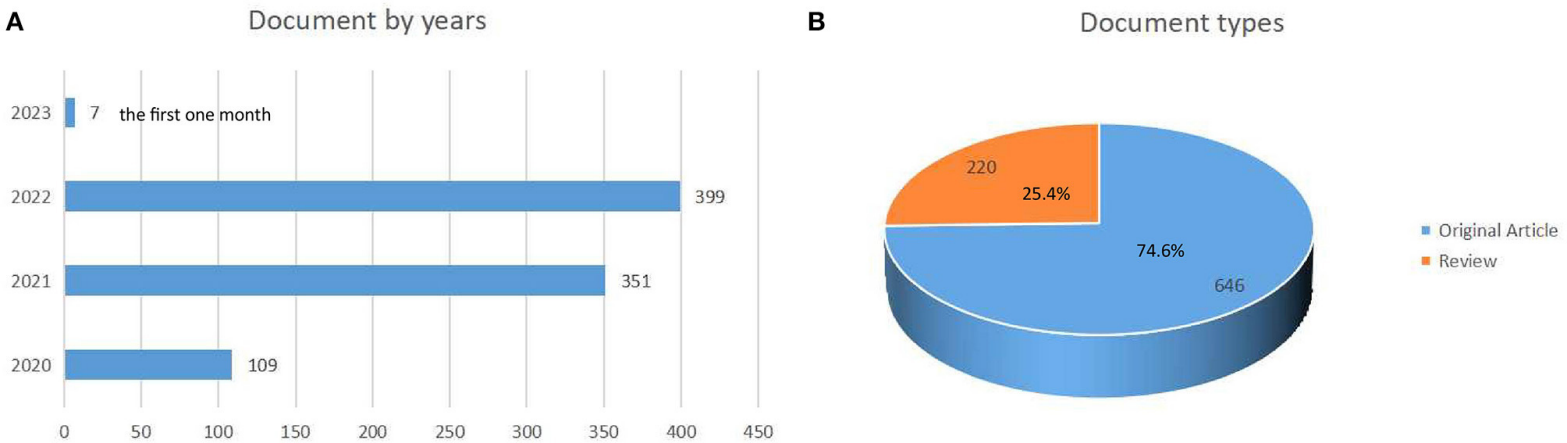
The year **(A)** the article is published. Type **(B)** of article published.

Figure 1 shows the flow chart. One thousand two hundred and eighty-nine articles were found in the initial search. After limiting the type of literature (original studies and reviews), noise words, and English language, 866 articles are retrieved. We also reviewed the following data: 7,691 TC, 8.88 CPP, and 36 H index. A total of 6,046 authors, 2,193 institutions, 93 countries/regions, and 424 journals contributed to these publications. From 109 publications in 2020 to 399 publications in 2022, as shown in Figure 2, seven were recorded in the 1st month of 2023. Then, 74.6% of the retrieved publications were original articles; 25.4% of the articles were reviewed.

### Top contributing authors

**Figure 3:**
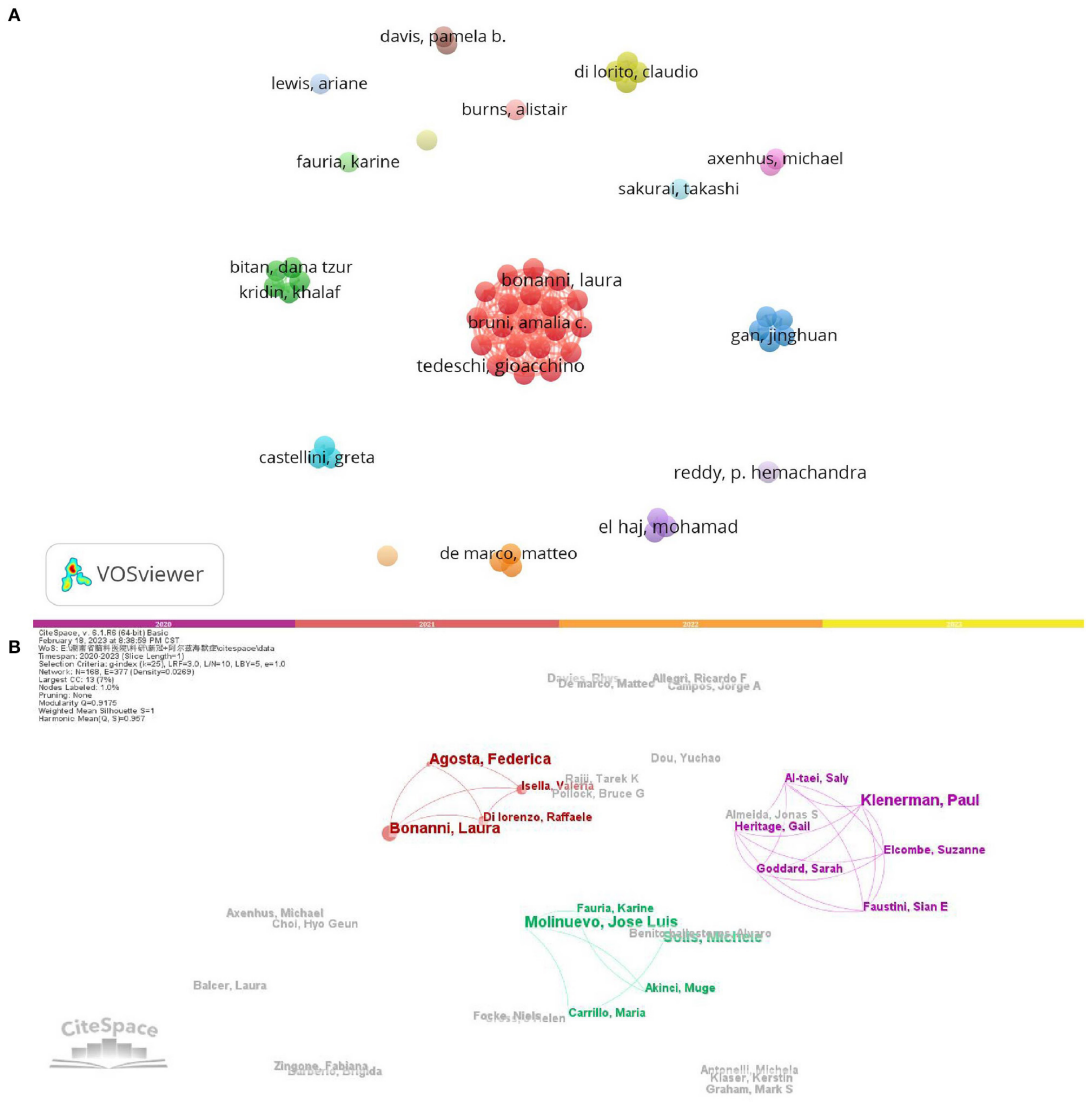
VOSviewer **(A)** and CiteSpace **(B)** show a collaborative network of prolific authors. Nodes represent the number of articles, while lines represent the tightness of the cooperative relationship.

Table 1 presents the authors who have published the most articles. Five authors tied for first place. Bonanni, Laura from Gabriele d'Annunzio University (Italy), Tedeschi, Gioacchino from the University of Campania Luigi Vanvitelli (Italy), Vanacore, Nicola from Natl Ctr Dis Prevent and Health Promot (Italy), Reddy, P. Hemachandra from Texas Tech University (USA) and El Haj, Mohamad from University of Nantes (France) were most prolific author. Each has published four articles. Furthermore, the author was identified as Bonanni, Laura from Gabriele d'Annunzio University (Italy; 197 TC and 49.25 CPP), followed by Tedeschi, Gioacchino from the University of Campania Luigi Vanvitelli (Italy; 184 TC and 46 CPP) and Vanacore, Nicola from Natl Ctr Dis Prevent and Health Promot (Italy; 183 TC and 45.75 CPP) with the highest citations. We use VOSviewer and CiteSpace software to analyze the author's collaborative network. In Figure 3A, there were 54 authors with more than three articles retrieved by VOSviewer. Similarly, in Figure 3B, these authors were also identified through CiteSpace. The respective active period was shown. Agosta, Federica, Allegri, Ricardo F were active in 2020, while Bonanni, Laura and, Focke, Niels were active in 2021; Klenerman, Paul and, Solis, Michele were active in 2022. Four collaborating scholars were identified, with more collaboration occurring in groups including Bonanni, Laura and, Tedeschi, and Gioacchino.

**Table 1:** Authors with the highest number of publications.

**Rank**	**Author**	**Publication**	**TC**	**CPP**	**Institution**	**Country**
1a	Bonanni, Laura	4	197	49.3	University G dAnnunzio	Italy
1b	El Haj, Mohamad	4	143	35.8	University Nantes	France
1c	Reddy, P. Hemachandra	4	94	23.5	Texas Tech University	USA
1d	Tedeschi, Gioacchino	4	184	46.0	University Campania Luigi Vanvitelli	Italy
1e	Vanacore, Nicola	4	183	45.8	Natl Ctr Dis Prevent and Health Promot	Italy

TC, total citations; CPP, citations per publication.

### Top contributing institutions

**Figure 4:**
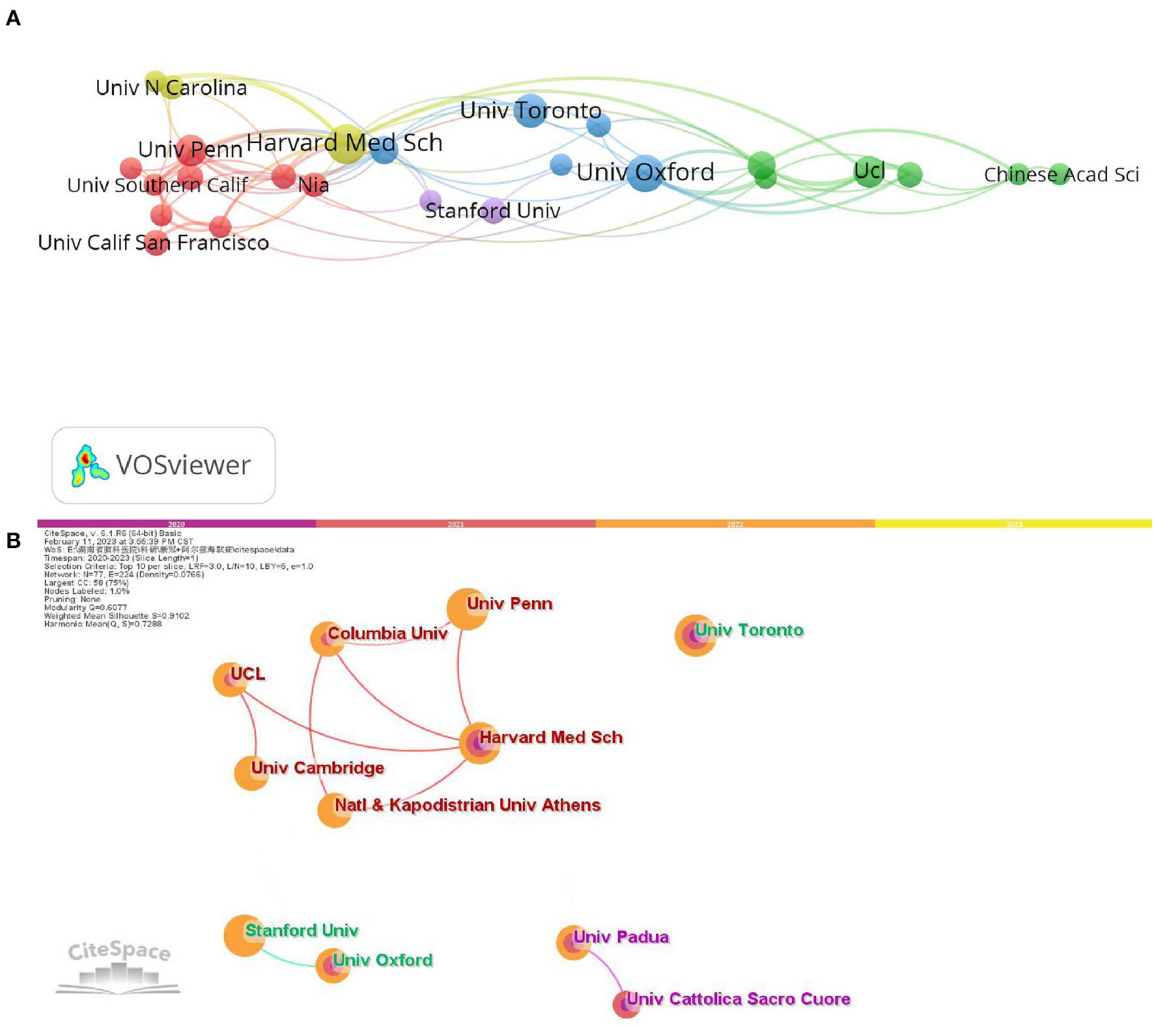
The network of cooperative relationships among institutions is shown through VOSviewer **(A)** and CiteSpace **(B)**. A node's size represents the number of articles the institution has published, and a line's width represents how close the institutions have collaborated.

The top 10 institutions with the most publications are listed in Table 2. The number of articles published by the top 10 institutions accounts for 14.4%. There were 17 papers published by Harvard Medical School in the United States, 16 by the University of Padova in Italy, and 15 by the University of Oxford in the United Kingdom. According to citations, the University of Toronto ranked first with 339 TC, followed by the University of Padua, Italy, with 264 TC, and the Universita Cattolica del Sacro Cuore with 187 TC. Institutional cooperation networks are visualized using VOSviewer and CiteSpace software. As shown in Figure 4A, VOSviewer identifies 35 institutions that have published at least seven papers, and the temporal evolution of these institutions is shown in Figure 4B. The University of Padua, the University of Pavia, and the Universita Cattolica del Sacro Cuore were active in 2020 but less active in 2021 and 2022. In terms of collaboration with other institutions, Harvard Medical School was the most prominent. Oxford University and King's College London are close behind.

**Table 2:** A list of the 10 most prolific institutions.

**Rank**	**Institution**	**Publication**	**TC**	**CPP**	**Country**
1	Harvard Med Sch	17	126	7.4	USA
2	Univ Padua	16	264	16.5	Italy
3	Univ Oxford	15	88	5.9	UK
4	Univ Toronto	13	339	26.1	Canada
5a	Ucl	12	107	8.9	UK
5b	Univ Penn	12	80	6.7	USA
6a	Kings Coll London	10	100	10	UK
6b	Univ Cattolica Sacro Cuore	10	187	18.7	Italy
6c	Univ Pavia	10	161	16.1	Italy
6d	Univ Washington	10	47	4.7	USA

TC, total citations; CPP, citations per publication.

### Top contributing countries

**Figure 5:**
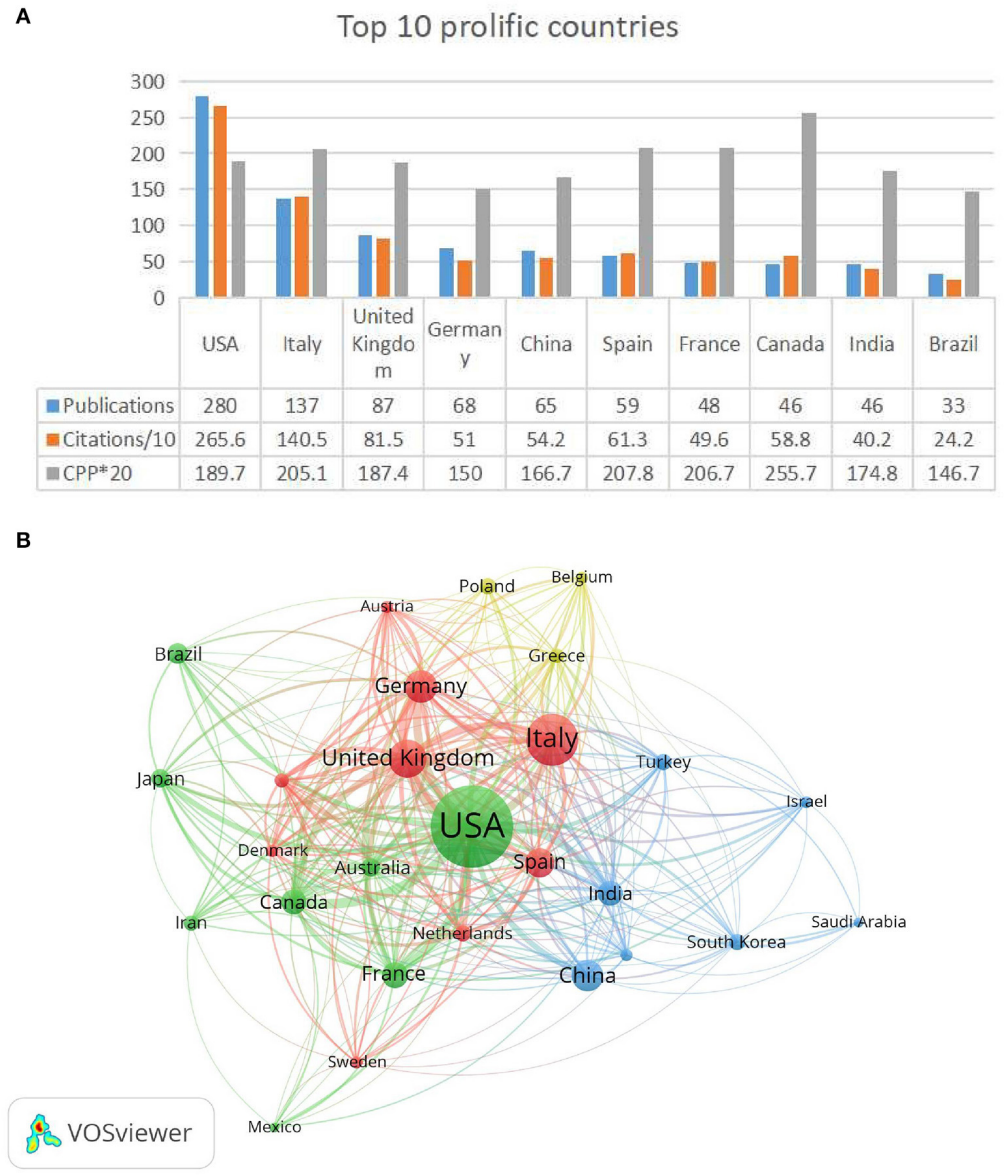
**(A)** A summary of the top 10 productive countries, as well as their publications, the number of citations ( × 0.1), and the number of citations per publication ( × 20). **(B)** Cooperation between States. A node's size indicates the number of articles, while the link's width indicates the degree of cooperation.

In Figure 5, the countries with the highest productivity were shown along with their respective collaborations. The results show that the United States has the highest productivity with 280 papers accounting for 32.3% of the total productivity with 2,656 TC, followed by Italy (137 papers, 1,405 TC) and the United Kingdom (87 papers, 815 TC; Figure 5A). According to CPP, Italy ranks sixth (*n* = 10.3), below Canada (*n* = 12.8), Scotland (*n* = 10.9), the Netherlands (*n* = 10.7), Spain (10.4), and France (10.33). Co-author country analysis in VOSviewer reveals collaboration between countries. At least 12 papers from 27 countries were selected for visualization, among which the nodes of the United States, the United Kingdom, Germany, Italy, and Canada were the most prominent. The links were relatively thick, indicating that their cooperation and academic influence were closer in this region (Figure 5B).

### Top contributing and co-cited journals

Table 3 shows the 10 most active publications on COVID-19 Alzheimer's disease papers ranked Q1, Q2, or Q3 in JCR. At number one on the list was the Journal of Alzheimer's Disease (*n* = 43), followed by Frontiers in Psychiatry (*n* = 25) and the International Journal of Molecular Sciences and Journal of Clinical Medicine (*n* = 20). Alzheimer's and Dementia has the most citations per publication (*n* = 68.7), followed by Frontiers in Psychiatry (*n* = 20.0) and Frontiers in Aging Neuroscience (*n* = 16.9). The Lancet ranks first (*n* = 836), followed by the New England Journal of Medicine (*n* = 788) and Nature (*n* = 670) in terms of co-citations.

**Table 3:** Top 10 for high output and co-cited journals.

**Rank**	**Journal**	**Publication**	**TC**	**CPP**	**IF**	**JCR**	**Co-cited journal**	**Co-citations**
1	Journal Of Alzheimer's Disease	43	710	16.5	4.2	Q2	Lancet	836
2	Frontiers In Psychiatry	25	501	20.0	5.4	Q2	New Engl J Med	788
3a	International Journal Of Molecular Sciences	20	130	6.5	6.2	Q1	Nature	670
3b	Journal Of Clinical Medicine	20	68	3.4	5.0	Q2	Plos One	667
4	Plos One	17	94	5.5	3.8	Q2	J Alzheimer's Dis	665
5	Frontiers In Neurology	15	203	13.5	4.1	Q2	Alzheimer's Dement	553
6	Frontiers In Aging Neuroscience	14	236	16.9	5.7	Q1	P Natl Acad Sci Usa	518
7a	Alzheimer's and Dementia	13	893	68.7	16.7	Q1	Neurology	516
7b	Brain Sciences	13	117	9	3.3	Q3	J Virol	465
7c	Frontiers In Public Health	13	46	3.5	6.5	Q1	Jama-J Am Med Assoc	464

TC, total citations; CPP, citations per publication; IF, the impact factor (2022); JCR, Journal Citation Reports (2022).

### Top cited articles

The top 10 most-cited publications are shown in Table 4. Of these, four papers discussed the impact of COVID-19 on patients with AD (9, 10, 13, 28). Three papers discuss the impact of the novel coronavirus-19 virus in the care of patients with Alzheimer's disease (29–31). A report on the effects of SARS-CoV-2 on the behavior and psychology of patients with AD (17). One paper described the impact of SARS-CoV-2 on neuropsychiatric symptoms and quality of life in patients with AD (19). One paper reported an increased prevalence of SARS-CoV-2 in patients with AD (32). Gaugler et al. published the most significant cited paper (457 TC) in Alzheimer's and Dementia. This article introduced the public health problems caused by Alzheimer's disease, including incidence and prevalence, mortality and disability, and medication and care costs.

**Table 4:** Top 10 cited articles.

**Rank**	**Title**	**First authors**	**Type**	**Citation**	**Journal**	**Year**
1 (29)	2021 Alzheimer's disease facts and Figures	Gaugler, J	Article	457	Alzheimer's and Dementia	2021
2 (13)	Anticipating and Mitigating the Impact of the COVID-19 Pandemic on Alzheimer's Disease and Related Dementias	Brown, EE	Article	238	American Journal of Geriatric Psychiatry	2020
3 (10)	Neurobiology of COVID-19	Fotuhi, M	Review	201	Journal of Alzheimer's Disease	2020
4 (28)	Long-Term Respiratory and Neurological Sequelae of COVID-19	Wang, FZ	Review	146	Medical Science Monitor	2020
5 (30)	2022 Alzheimer's disease facts and Figures	Gaugler, J	Article	113	Alzheimer's and Dementia	2022
6 (17)	Behavioral and Psychological Effects of Coronavirus Disease-19 Quarantine in Patients With Dementia	Cagnin, A	Article	108	Frontiers in Psychiatry	2020
7 (31)	Living with dementia: increased level of caregiver stress in times of COVID-19	Cohen, G	Article	106	International Psychogeriatrics	2020
8 (19)	Neuropsychiatric Symptoms and Quality of Life in Spanish Patients with Alzheimer's disease during the COVID-19 Lockdown	Lara, B	Article	104	European Journal of Neurology	2020
9 (9)	A systematic review of neurological symptoms and Complications of COVID-19	Chen, XL	Review	103	Journal of Neurology	2021
10 (32)	COVID-19 and dementia: Analyses of risk, disparity, and outcomes from electronic health records in the US	Wang, QQ	Article	88	Alzheimer's and Dementia	2021

Further, it explained the impact of the novel coronavirus-19 overall effect (29). Brown et al. wrote the second most-cited paper (238 TC). They were published in the American Journal of Geriatric Psychiatry. In this special article, the authors examined the current and expected impact of the pandemic on individuals with AD (13).

With 201 total citations, Fotuhi et al. produced the third-highest citation count. This paper was published in the Journal of Alzheimer's Disease. The authors reviewed some of the acute neurological symptoms of patients with SARS-CoV-2 and the impact of SARS-CoV-2 on Alzheimer's disease (10).

### Analysis of co-citation references

**Figure 6:**
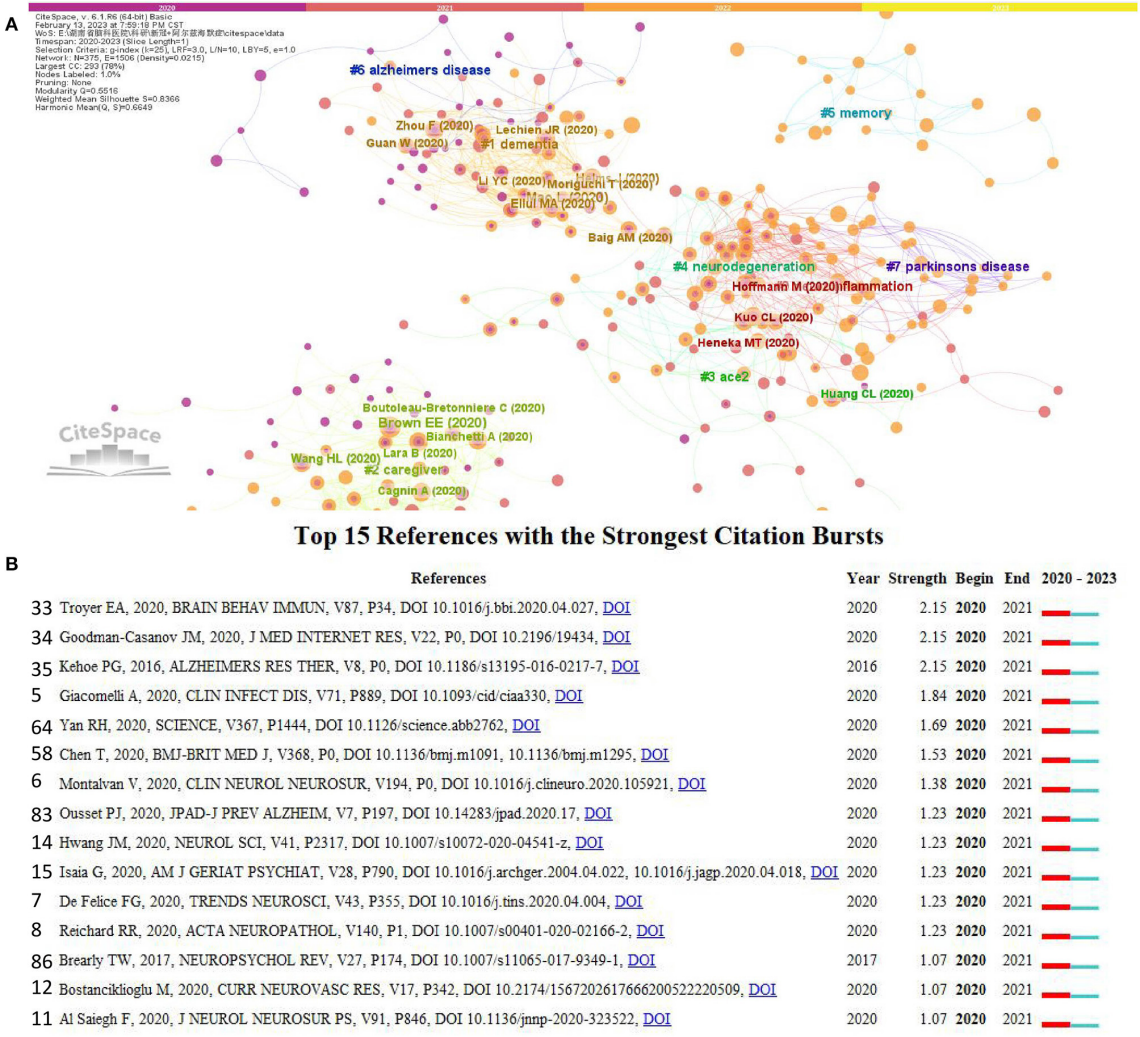
**(A)** CiteSpace aggregates reference co-citations. Darker colors indicate earlier co-occurrence citation relations between nodes and links. Nodes in a network named by the first author (year of publication) contain references cited at least 30 times. Citations are positively correlated with the size of the node. The red text indicates the cluster name automatically recognized by the Citespace LLR algorithm. **(B)** The top 15 most explosive references. Red bars indicate burst duration. The burst strength indicates that this paper studies the importance of the field.

We performed the analysis of co-citation references with CiteSpace. We found an evolution of thematic structure in COVID-19 Alzheimer's disease research. There were at least 30 citations in this network for these articles, as shown in Figure 6A. Articles published in 2020 were structured around the themes of “Alzheimer's disease,” “ACE-2(angiotensin-converting enzyme-2),” “caregiver,” and “dementia.” Namely “neurodegeneration,” which was the primary concern in 2021. The article published in 2022 was about “memory.” In addition, citation bursts were used to identify significant references that contributed to knowledge in the field. CiteSpace identified the top 15 most-cited publications (Figure 6B). Three articles have the highest citation bursts (*n* = 2.15). Troyer et al. published an article in Brain, Behavior, and Immunity on April 13, 2020. In this article, the authors compare previous studies' findings on neuropsychiatric symptoms associated with coronavirus infection. This paper also discussed its possible pathogenic mechanism, including virological and immunological evidence (33). Another article was by Goodman-Casanov et al., published in the Journal of Medical Internet Research on April 17, 2020. This article reported their findings during COVID-19 confinement, which show that most of our vulnerable population's bodies, minds, and wellbeing were at their best. However, people who lived alone reported more negative mental reactions and had more sleep problems (34). Finally, Kehoe et al. published them in Alzheimer's Research and Therapy on November 25, 2016, which reports that ACE-2 activity was reduced in AD and was an essential regulator of the central classical ACE-1 (angiotensin-converting enzyme-1)/Ang II (Angiotensin II)/AT1R (angiotensin II type 1 receptor) axis of the RAS (renin-angiotensin system). Also, the dysregulation of this pathway likely plays a significant role in the pathogenesis of AD (35).

### Analysis of keywords

**Figure 7:**
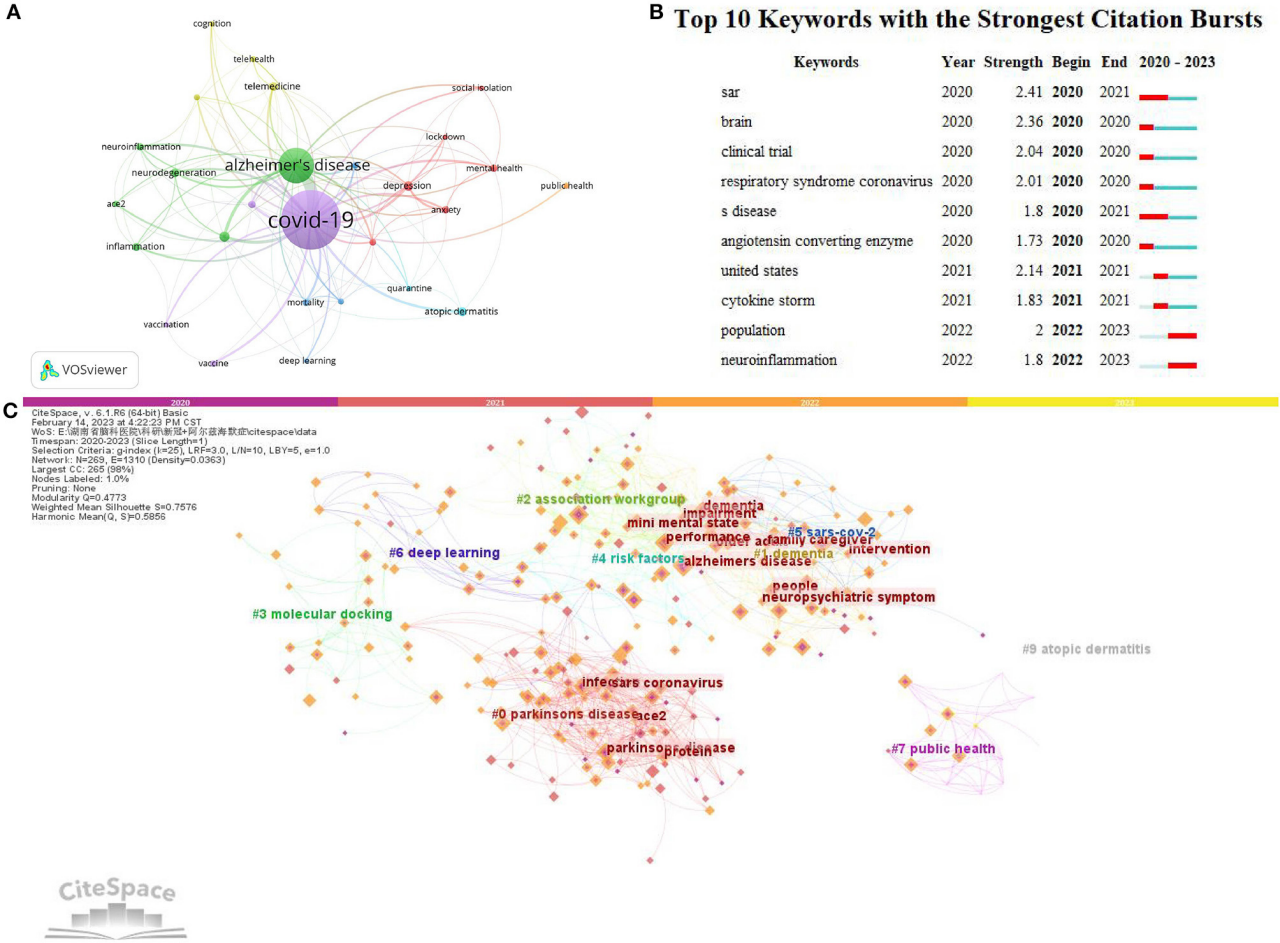
Author keyword analysis. **(A)** The VOSviewer visualizes keyword co-occurrence networks. Large nodes represent high-frequency keywords; A closer relationship is indicated by the same color; **(B)** Top 10 most cited keywords. Red bars indicate burst persistence Time. The stronger the keyword, the more important it is. **(C)** From 2020 to 2023 by CiteSpace keyword clusters are named by the LLR algorithm.

Using VOSviewer and CiteSpace, this paper analyzes the co-occurrence of keywords in the network environment to identify hot topics and possible development directions. In addition, we also use dictionaries (Supplementary Table S1 was provided in the file Supplementary material) to incorporate keywords with similar semantics. COVID-19 replaces SARS-CoV-2, for instance. VOSviewer identified 2,405 author keywords. Co-occurrence network-only keywords with more than 11 occurrences were visualized. Finally, the 27 keywords are divided into seven color clusters (Figure 7A). The top nine most frequently used keywords were “COVID-19” (*n* = 522), “Alzheimer's disease” (*n* = 222), “Parkinson's disease” (*n* = 28), “depression” (*n* = 25), “atopic dermatitis” (*n* = 23), “neurodegeneration” (*n* = 23), “telemedicine” (*n* = 23), “inflammation” (*n* = 21), and “cognitive impairment” (*n* = 19). The burst module in CiteSpace can determine the most frequently used keywords over time. Figure 7B shows the 10 keywords with the most substantial citation outbreak, of which “sar” (SARS-CoV-2) was the most frequent (*n* = 2.41), followed by “brain” (*n* = 2.36) and “the United States” (*n* = 2.14). In addition, this paper also uses the keyword coexistence analysis in Citespace to observe it in time series. The possible development trend in this aspect is analyzed. According to their average publication year, Figure 7C shows the keyword root. Dark colors (e.g., purple and scarlet) indicate early trending keywords, namely, “COVID-19,” “Alzheimer's disease,” “depression,” “neuropsychiatric symptom,” “telemedicine,” “social isolation,” “Parkinson's disease,” “deep learning,” “brain,” and “inflammation.” Colors representing recent popular keywords include saffron and yellow, such as “neuroinflammation,” “molecular mechanism,” “neurodegenerative disorder,” and “cognitive function.”

## Discussion

The COVID-19 pandemic was disrupting the world and its healthcare systems in unprecedented ways, with direct risks and implications for people with AD that cannot be ignored (13). During the SARS-CoV-2 pandemic, many articles related to this issue have been published, as can be seen from its dramatic growth rate—nearly four times as many papers were published in 2022 as in 2020.

During this period, the virus mutated. Many variants have emerged, among which the main lineages pose the greatest global threat (36). Emerging Omicron variants and their lineages have led to a rapid and substantial increase in COVID-19 cases globally while adversely affecting the protective efficacy of existing vaccines and antibody-based therapies (37). This is worse for AD patients. Therefore, expect more articles describing SARS-CoV-2′s impact on AD.

### Author analysis

Bonanni, Laura from Gabriele d'Annunzio University (Italy), Tedeschi, Gioacchino from the University of Campania Luigi Vanvitelli (Italy), Vanacore, Nicola from Natl Ctr Dis Prevent and Health Promot (Italy), Reddy, P. Hemachandra from Texas Tech University (USA) and El Haj, Mohamad from the University of Nantes (France) were the most prolific authors. Each of them published four articles. In terms of total citations and citations per publication, Bonanni, Laura from Gabriele d'Annunzio University (Italy; 197 TC and 49.25 CPP) and Tedeschi, Gioacchino from the University of Campania Luigi Vanvitelli (Italy; 184 TC and 46 CPP) ranked first and second, respectively. This result shows that new scholars can understand how to conduct influential research and grasp hot issues in this field by reading their works. For example, in 2020, Bonanni, Laura, Tedeschi, Gioacchino, Vanacore, Nicola, and El Haj, Mohamad, focused on the behavioral and psychological effects of COVID-19 on AD patients (17, 18, 38), and in 2021 and 2022, the focus shifts to the psychiatric impact of COVID-19 on people with AD and family caregivers (20, 39–42) as well as the impact of psychological interventions on caregivers (43) and the risk of vaccination for people with AD (44). Reddy focused on the relationship between COVID-19 and the neural mechanisms (45), immunity (46), and clinical manifestations (47) of AD.

### Country and institution analysis

The field of Alzheimer's disease research for SARS-CoV-2 has received extensive attention from researchers in 93 countries/regions. Seven of the 10 most productive countries are developed, and three (China, India, and Brazil) are developing. The per capita GDP of developed countries is higher than that of developing countries, and they will care more about their physical health. In addition, developed countries were also likely to increase their prevention and research on the new coronavirus. As a result of COVID-19′s high transmission rate, developing countries still face severe challenges. This project proposes that research on Alzheimer's disease should be increased to promote the healthy development of developing countries in this area. In addition, most authors and institutions that published articles are from Italy (3/5 authors and 3/10 institutions) and the United States (1/5 authors and 3/10 institutions).

The first European country to impose a national lockdown was Italy, a strict lockdown that prevented people from leaving their homes for all but basic activities. Alzheimer's disease (AD) is a disease that seriously endangers human health. As the elderly population grows, Alzheimer's disease will increase (48). In Italy, 11.9% of people who died from COVID-19 between May and September 2020 had AD or dementia (49). It follows that Italy invested much money, human resources, and material in AD research during the SARS-CoV-2 pandemic. Most of the most cited authors were also from Italy. However, the most cited institutions were in Canada, followed by Italy. This may be related to the prevalence of AD. In Canada, the prevalence of Parkinson's disease and AD increases dramatically as people age past 65 due to longer life expectancy and increased incidence, both of which are expected to double in the next 20 years (50).

### Journal analysis

The top 10 high-yield journals (Q1/Q2) found in the survey rank the highest in this field; according to Journal Citation Reports 2022 (JCR), only one was Q3. In addition to the number of published papers, journals were also evaluated based on the number of citations per publication. In front of the ten productive journals, only Alzheimer's and Dementia (*n* = 68.7), Frontiers in Psychiatry (*n* = 20.0), Frontiers In Aging Neuroscience (*n* = 16.9), and Journal of Alzheimer's Disease (*n* = 16.5) exceeded the average citations per publication of all publications (*n* = 16.36). The remaining journals were cited less frequently in the field. To represent the field's most classical and influential journals, this paper lists the top 10 most co-cited journals, such as the Lancet and the New England Journal of Medicine. Understanding prolific journals can help researchers choose publishers to submit articles to and master new topics. In addition, publication in co-cited journals can increase literature knowledge for future works.

### Hotspots and research trends

The relevant knowledge base can be obtained by analyzing keywords and references. Using VOSviewer and CiteSpace, the article analyzes keywords and related literature related to COVID-19 research in AD. The results of the study show that the research objects were constantly changing. For example, in 2020, articles were centered on “Alzheimer's disease,” “COVID-19,” “risk factors,” “care,” and “Parkinson's disease,” while in 2021, the topics changed to “neurodegenerative diseases,” “cognitive impairment,” and “older adults.” In 2022, the topics changed to “neuroinflammation,” “spike protein,” “quality of life,” and “neurological complications.” The following sections build a cluster-based augmented keyword and citation analysis and have a brief discussion.

Mental health is represented by cluster 1 (red in Figure 7A).

The primary keywords were “anxiety,” “depression,” “lockdown,” “mental health,” “mild cognitive impairment,” and “social isolation,” which were shown in cluster #5 in CiteSpace. In most countries, the core of COVID-19 restrictions was physical distancing from others, or even “self-isolation” or “isolation.” There is strong evidence that being isolated from others damages mental health (51–53). During the early days of the SARS-CoV-2 pandemic, researchers focused on behavioral and psychological problems associated with AD (Figure 6A). For example, the four most cited authors (Bonanni, Laura, Tedeschi, Gioacchino, Vanacore, Nicola, and El Haj, Mohamad) focused on the behavioral and psychological effects of COVID-19 and related factors on patients with AD. They reported that isolation caused a rapid increase in behavioral and psychological symptoms in about 60% of patients (17). Confinement appears to affect neuropsychiatric symptoms and increase levels of depression in Alzheimer's disease patients with low baseline cognitive function (18, 41). For Alzheimer's disease (AD) patients living in nursing homes in France, depression and anxiety were higher after the COVID-19 crisis than before (38). In addition, they highlight the mental impact of SARS-CoV-2 and related factors on family caregivers and their caregivers. Stress was prevalent among family caregivers of people with Alzheimer's disease during the SARS-CoV-2 pandemic (40).

Other researchers have reported this as well. Other researchers have reported this as well. In Argentina, COVID-19 confinement increased stress on caregivers independent of the dementia stage of Alzheimer's disease. However, those caring for severe cases were more stressed than those caring for milder disease forms (31). In addition, researchers compared neuropsychiatric symptoms in older adults with and without Alzheimer's dementia. The available data show that the novel coronavirus epidemic has caused great adverse consequences for humans, including the mental health of older people with and without dementia (54). There was a significant positive correlation between loneliness due to social isolation and anxiety, depression, and trauma-related distress in older adults (55). A Spanish study showed that patients with mild cognitive impairment and AD-induced dementia had negative changes in neuropsychiatric symptoms, particularly apathy (19).

Cluster 2 represents Alzheimer's disease and its associated neural mechanisms.

The primary keywords were “ACE-2,” “Alzheimer's disease,” “inflammation,” “neurodegeneration,” “neuroinflammation,” and “Parkinson's disease,” which were shown in cluster #0 in CiteSpace. An estimated 6.2 million (in 2021) and 6.5 million (in 2022) Americans age 65 and older will have Alzheimer's dementia (29, 30). Alzheimer's disease (AD) has become a significant complication of SARS-CoV-2 (56, 57). In high-risk patients, COVID-19 infection can cause pulmonary and systemic inflammation and damage to multiple organs (58). Neuroinflammation was one of the pathogenesis of AD. Neuroinflammation contributes as much to the pathogenesis of AD as accumulated plaques in old age or passive systems activated by NFT (neurofibrillary tangles). Systemic inflammation also affects cognitive function and contributes to the progression of neurodegenerative diseases (59). A common neuroinflammatory response exists between COVID-19 and AD (60). ACE-2 (Angiotensin-converting enzyme 2) was related to AD. The brain's neurons, glial cells, endothelial cells, and smooth muscle cells expressed ACE-2. ACE-2 was also expressed in the temporal lobe and hippocampus. The pathogenesis of AD was linked to these brain regions (61). ACE-2 expression levels increase with the severity of AD, according to studies (62, 63). The mechanism of neural invasion by SARS-CoV-2 may be related to ACE-2 (28, 64). It has been hypothesized that COVID-19 may target ACE-2 and inhibit its expression or activity, leading to cognitive dysfunction and exacerbating cognitive dementia in AD patients (56). “Inhibitors” of ACE-2 have been considered “potential” treatments for “neurodegenerative disorders” such as “AD” (65).

Cluster 3 represents deep learning and its applications (blue in Figure 7A).

The primary keywords were “caregivers,” “deep learning,” “mortality,” and “risk factors,” which were shown in cluster #6 in CiteSpace. Deep learning (DL) is a computer technology branch of machine learning. This was a neural network-based algorithm for data expression learning. It can learn directly from raw data and use output layers with multiple hidden layers (66). Convolutional neural networks (CNN), a kind of deep learning, have succeeded in diagnosing AD because they can automatically extract features (67, 68). Deep learning and CNN, in particular, can help doctors and patients remotely check for AD, determine the stage of AD based on the AD spectrum, and provide recommendations for patients based on their AD stage (69). Al-Adhaileh et al. also reported that deep learning could be used to classify and recognize AD (70). Interestingly, deep learning can also be applied to COVID-19 patients to help diagnose COVID-19 (71). One study estimated that 19% of 260 COVID-19 patients with AD died (32). SARS-CoV-2 and AD share similar risk factors, such as advanced age (72–74). Deep learning has been shown to predict SARS-CoV-2 mortality in patients with Alzheimer's, identify risk factors, and relate to the pathophysiological processes of human disease. This method can efficiently transform a large amount of medical and biomedical information, to improve people's physical quality (75).

In Figure 7A, cluster 4 represents telemedicine (yellow).

The primary keywords were “cognition,” “neurodegenerative diseases,” “telehealth,” and “telemedicine,” which were shown in cluster #1 in CiteSpace. Telemedicine, a term introduced in the 1970's, is a medical procedure in which a doctor and patient do not physically interact through an interactive multimedia communication system. Telemedicine refers to the application of information and communication technology (ICT) to medical research and provides remote assistance to patients through expert networks or information exchange between experts and patients (76). Telemedicine could be used in neurodegenerative diseases, including Alzheimer's and Parkinson's (77). Tele-rehabilitation was also of note to us, which will make it possible for patients with neurodegenerative diseases to have access to cognitive rehabilitation in all situations where the patient and therapist are not in the same location due to specific limitations (such as the SARS-CoV-2 pandemic) (78). Gosse et al. also reported that telemedicine might improve access to health care for people with AD and related dementias, especially during the COVID-19 pandemic (79–81). Telemedicine can significantly improve all aspects of care for people with Alzheimer's and dementia (82, 83). Telemedicine can also be applied to conditions like severe asthma (84, 85). Telemedicine also has good practice for neuropsychological assessment (86). Telemedicine was also widely used in military medical services (87). In emergencies such as coronavirus infections, telemedicine is a feasible, safe, and effective means of health service (88). While telemedicine can help improve access to health care, patients and caregivers see significant limitations compared to face-to-face services (89).

Cluster 5 represents vaccine-related issues (purple in Figure 7A).

The primary keywords were “cognitive impairment,” “COVID-19,” “vaccination,” and “vaccine.”

In the context of COVID-19, many people have been vaccinated. Patients can develop breakthrough COVID-19 infections despite vaccination (90). However, vaccination can prevent severe morbidity and mortality from SARS-CoV-2 in the general population (91–93). SARS-CoV-2 patients are at high risk of developing Alzheimer's disease and need to be vaccinated to reduce the prevalence of SARS-CoV-2 (94). There was evidence that SARS-CoV-2 vaccination was safe for adolescents (95). The SARS-CoV-2 vaccine was also safe and well-tolerated in patients with cognitive impairment (44). Studies have shown that COVID-19 vaccines are generally safe overall, but reported adverse reactions may vary by age and gender (96). Surprisingly, some people refuse to be vaccinated (97). One reason could be adverse reactions to vaccinations (98–101).

### Limitations

This study has two limitations. First, based on the literature scale in VOSviewer and CiteSpace, the relevant data of COVID-19-related Alzheimer's disease patients were all used in the same database (WOSCC), and there was a selection bias. Other sources of data, such as PubMed and Scopus, support only one bibliographic measurement tool, such as VOSviewer. For this reason, this project intends to use CiteSpace and VOSviewer to analyze multiple data sets to reduce selection errors and avoid the analysis of duplicate data existing in multiple data sets. Secondly, since only papers published in English were included, language discrimination will also have a particular impact on this study. Future investigations should be combined with other language literature to draw broad conclusions.

## Conclusion

The analysis in this article outlines an overview of COVID-19 in AD. During the SARS-CoV-2 pandemic, topics such as “mental health,” “Alzheimer's disease and its associated neural mechanisms,” “deep learning and its applications,” “telemedicine,” and “vaccine-related issues” have attracted considerable attention. There were a few suggestions: (1) During the COVID-19 pandemic, especially with quarantine measures, special attention should be paid to the mental health of AD patients and their family caregivers, giving them as much attention as possible; (2) In the context of the SARS-CoV-2 pandemic, it will be inevitable for AD patients to be complicated with COVID-19. Research on the neural mechanisms related to them may be helpful for our treatment. Among them, the research on neuroinflammation and ACE-2 was active, which will be the direction of our consideration; (3) deep learning can contribute to the early diagnosis of AD and COVID-19, which may be the trend of our research in the future; (4) we can build more on telemedicine as a viable, safe, and efficient healthcare tool in exceptional circumstances; (5) vaccination is generally safe and can reduce severe morbidity and mortality. We should increase vaccine research, reduce adverse reactions, dispel public concerns, and protect people's lives and health.

## Data availability statement

The original contributions presented in the study are included in the article/Supplementary material, further inquiries can be directed to the corresponding authors.

## Author contributions

CC and SL conceived the study, participated in the design, and drafted the manuscript. GZ and CX participated in data collection and interpretation. XC and HQ were responsible for redoing the chart. XL and YL compiled the literature and participated in the discussion. HC and CB were responsible for guiding the whole process. All authors contributed to the article and approved the submitted version.
